# Enzyme-Mediated Quenching of the *Pseudomonas* Quinolone Signal (PQS) Promotes Biofilm Formation of *Pseudomonas aeruginosa* by Increasing Iron Availability

**DOI:** 10.3389/fmicb.2016.01978

**Published:** 2016-12-09

**Authors:** Beatrix Tettmann, Christine Niewerth, Frank Kirschhöfer, Anke Neidig, Andreas Dötsch, Gerald Brenner-Weiss, Susanne Fetzner, Joerg Overhage

**Affiliations:** ^1^Karlsruhe Institute of Technology, Institute of Functional InterfacesKarlsruhe, Germany; ^2^Institute for Molecular Microbiology and Biotechnology, University of MünsterMünster, Germany

**Keywords:** *Pseudomonas aeruginosa*, *Pseudomonas* quinolone signal, quorum sensing, quorum quenching, biofilm, PQS dioxygenase

## Abstract

The 2-alkyl-3-hydroxy-4(1*H*)-quinolone 2,4-dioxygenase HodC was previously described to cleave the *Pseudomonas* quinolone signal, PQS, which is exclusively used in the complex quorum sensing (QS) system of *Pseudomonas aeruginosa*, an opportunistic pathogen employing QS to regulate virulence and biofilm development. Degradation of PQS by exogenous addition of HodC to planktonic cells of *P. aeruginosa* attenuated production of virulence factors, and reduced virulence *in planta*. However, proteolytic cleavage reduced the efficacy of HodC. Here, we identified the secreted protease LasB of *P. aeruginosa* to be responsible for HodC degradation. In static biofilms of the *P. aeruginosa* PA14 *lasB*::Tn mutant, the catalytic activity of HodC led to an increase in viable biomass in newly formed but also in established biofilms, and reduced the expression of genes involved in iron metabolism and siderophore production, such as *pvdS, pvdL, pvdA*, and *pvdQ*. This is likely due to an increase in the levels of bioavailable iron by degradation of PQS, which is able to sequester iron from the surrounding environment. Thus, HodC, despite its ability to quench the production of virulence factors, is contraindicated for combating *P. aeruginosa* biofilms.

## Introduction

*Pseudomonas aeruginosa* is one of the most important opportunistic human pathogens, causing a variety of life-threatening infections in immunocompromised patients (Gellatly and Hancock, 2013). Moreover, *P. aeruginosa* is the dominant and most significant pathogen in patients suffering from cystic fibrosis, causing very difficult to treat pulmonary infections (Hutchison and Govan, 1999; Rajan and Saiman, 2002). It has a number of intrinsic resistance mechanisms including multidrug resistance efflux systems, low outer membrane permeability, and β-lactamases, produces a large arsenal of virulence factors, and moreover forms robust biofilms (Gellatly and Hancock, 2013).

Like many other pathogenic bacteria, *P. aeruginosa* co-ordinates group behavior, such as the synthesis of many virulence factors and biofilm development, via cell-to-cell communication or quorum sensing (QS) (Williams et al., 2007). The sophisticated QS network of *P. aeruginosa* comprises several interconnected signaling circuits, with the Las and Rhl systems producing and responding to *N*-3-oxo-dodecanoyl homoserine lactone and *N*-butanoyl homoserine lactone, respectively, and the PQS circuit using specific 2-*n*-alkyl-4(1*H*)-quinolone (AQ) signals. 2-Heptyl-3-hydroxy-4(1*H*)-quinolone, the “*Pseudomonas* quinolone signal” (PQS), is the major AQ signal in *P. aeruginosa* (Pesci et al., 1999). Both PQS and its biosynthetic precursor HHQ (2-heptyl-4(1*H*)-quinolone) act as coinducers of the transcriptional regulator PqsR (MvfR) (Déziel et al., 2005; Wade et al., 2005; Xiao et al., 2006; Diggle et al., 2007). PQS, besides its role as a QS signal molecule, modulates membrane properties (Mashburn and Whiteley, 2005), acts as ferric iron chelator (Bredenbruch et al., 2006; Diggle et al., 2007) and pro-oxidant (Häussler and Becker, 2008), and exerts pro-apoptotic and host immune modulatory activities (Hooi et al., 2004; Skindersoe et al., 2009; Hänsch et al., 2014).

PQS has been reported to promote biofilm development (Diggle et al., 2003), and *pqsA* and *pqsC* mutants, which are unable to produce AQs, are poor biofilm producers (Müsken et al., 2010). Because PQS did not induce biofilm formation in mutants deficient of the signal transduction histidine kinase RetS or the sensor/response regulator protein GacS, it has been suggested that enhancement of biofilm by PQS is at least partially dependent on the RetS-GacAS-Rsm system (Guo et al., 2014). GacA positively controls the small regulatory RNA RsmZ, which acts by sequestering the RsmA protein (Sonnleitner and Haas, 2011). RsmA directly and via modulating cyclic di-GMP levels controls diverse functions related to the *P. aeruginosa* switch between planktonic and biofilm lifestyles (Frangipani et al., 2014).

Because PQS signaling is involved in control of virulence factor production as well as biofilm maturation, interference with this QS system has been discussed as an attractive anti-virulence strategy (Lesic et al., 2007; Pustelny et al., 2009; Storz et al., 2012; Lu et al., 2014). Possible targets to interfere with QS circuits are the enzymes involved in signal biosynthesis, the signal receptor, or the signal itself. With respect to the latter, it seems that enzyme-catalyzed modification or degradation of bacterial signal molecules is wide-spread in nature. However, the majority of the quorum quenching enzymes identified to date are lactonases or acylases hydrolyzing *N*-acylhomoserine lactones, while only few enzymes have been described that are active toward other signal molecules (Fetzner, 2015). Among these, the heterocyclic-ring-cleaving enzyme “Hod” (1*H*-3-hydroxy-4-oxoquinaldine 2,4-dioxygenase) from *Arthrobacter* sp. Rue61a, despite its preference for 2-alkyl-3-hydroxy-4(1*H*)-quinolones with short alkyl substituents, is capable of cleaving PQS to form carbon monoxide and *N*-octanoylanthranilic acid. Exogenous addition of the enzyme to *P. aeruginosa* planktonic cultures resulted in significant down-regulation of the expression of key virulence factors and in reduction of *P. aeruginosa* pathogenesis in a plant infection model, highlighting the potential of quenching virulence through the enzymatic degradation of PQS (Pustelny et al., 2009). However, Pustelny et al. (2009) also observed that cleavage of Hod by extracellular proteases of *P. aeruginosa* reduced its efficiency as a quorum-quenching agent. *P. aeruginosa* secretes multiple proteases which degrade both soluble and structural host proteins and thus contribute to its pathogenicity. Among them, especially the metalloproteinases LasA, LasB, AprA and the serine protease PrpL (protease IV) have been correlated with virulence (Caballero et al., 2001).

In this study, we investigated the effect of HodC on biofilms of *P*. *aeruginosa* PA14 strains deficient in the production of the protease LasB, which was found to be liable for proteolytic degradation of the enzyme. The presence of exogenous, catalytically active HodC led to increased biomass in newly formed but also established biofilms, and down-regulated a set of genes which are under control of the ferric uptake regulator (Fur). Degradation of the iron chelator PQS is likely accompanied by an increase in readily bioavailable iron which is responsible for a gain in biofilm biomass.

## Materials and methods

### Bacterial strains, media, and culture conditions

The bacterial strains used in this study are listed in Table 1. All mutant strains were confirmed by PCR (data not shown). Growth was routinely performed in lysogeny broth (LB) or BM2 minimal medium (Overhage et al., 2008) at 37°C with shaking at 170 rpm unless otherwise indicated. When required, gentamicin was used at a final concentration of 30 μg/ml for *P. aeruginosa* transposon mutants and ampicillin and kanamycin at final concentrations of 100 and 30 μg/ml, respectively, for recombinant *Escherichia coli*.

**Table 1:** **Bacterial strains used in this study**.

**Strain**	**Description and characteristics^a^**	**References**
***P. aeruginosa***		
PA14	*Pseudomonas aeruginosa* PA14 wild-type	Rahme et al., 1995
*lasA*::Tn	PA14 transposon insertion mutant, ID 35267, Gm^r^	Liberati et al., 2006
*lasB*::Tn	PA14 transposon insertion mutant, ID 31938, Gm^r^	Liberati et al., 2006
*aprA*::Tn	PA14 transposon insertion mutant, ID 23768, Gm^r^	Liberati et al., 2006
*prpL*::Tn	PA14 transposon insertion mutant, ID 37740, Gm^r^	Liberati et al., 2006
***E. coli***		
M15 [pREP4, pQE30-*hodC*_H251A]	Recombinant strain for overexpression of HodC_H251A (iHodC)	Frerichs-Deeken et al., 2004
BL21 (DE3) [pET-23a-*hodC*]	Recombinant strain for overexpression of HodC	This work

a *Antibiotic resistance phenotype: Gm^r^, gentamicin resistance*.

For recording growth curves, bacterial cultures were grown overnight in LB and diluted in fresh LB to obtain starting optical densities of OD_600nm_ = 0.1. Aliquots of these dilutions were apportioned in 96-well microtiter plates (100 μl per well). Growth was recorded using a TECAN Infinite® 200 PRO plate reader (Tecan, Maennedorf, Switzerland) under shaking conditions. Two independent experiments were performed with three replicates for each strain or condition.

### HodC purification and activity assay

Since wild-type Hod protein is prone to oxidative dimerization by the formation of an intermolecular disulfide bridge, Hod protein carrying a substitution of Cys69 by serine, termed HodC, was used. HodC shows the same catalytic activity as Hod (Frerichs-Deeken et al., 2004). HodC-H251A protein, termed iHodC, which is virtually inactive due to its inability to initiate catalysis by deprotonating the organic substrate (Frerichs-Deeken et al., 2004), was used in control experiments. Purification of the recombinant His_6_-tagged HodC proteins from recombinant *E. coli* strains (Table 1) was performed as described by Beermann et al. (2007). For storage at −80°C, 10% glycerol (vol/vol) was added to the protein stock solutions. To control the activity of HodC protein before and after an experiment, its catalytic activity was determined spectrophotometrically by measuring 3-hydroxy-2-methyl-4(1*H*)-quinolone consumption as described previously (Frerichs-Deeken et al., 2004). 1 Unit of enzyme activity is defined as the amount of HodC catalyzing the conversion of 1 μmol substrate per minute under the conditions of the assay. Concentrations of HodC were determined by absorption measurements using an extinction coefficient (ε_280nm_) of 1.937 ml mg^−1^ cm^−1^ (Beermann et al., 2007).

### Enzyme stability assay

Stability of HodC against different proteases produced by *P*. *aeruginosa* was monitored by measuring the enzyme activity in the presence of stationary phase culture supernatants of *P*. *aeruginosa* PA14, and of the PA14 mutants *lasB*::Tn, *lasA*::Tn, *prpL*::Tn, and *aprA*::Tn, respectively. Bacterial strains were grown in LB medium overnight at 37°C and 160 rpm. These cultures were used to inoculate 15 ml LB medium to an OD_600 nm_ of 0.05, and cells were further grown for 10 h at 37°C and vigorous shaking. Afterwards, cells were pelleted by centrifugation (9000 × g, 10 min, 4°C) and the culture supernatants were collected and filter sterilized. For analysis, 100 μl/ml culture supernatant was incubated in sodium phosphate buffer pH 8.0 with 0.75 μg/ml HodC at 37°C, and enzyme activity was measured at different time points.

### Chemicals

The PQS cleavage product, *N*-octanoylanthranilic acid, was synthesized according to the method described by Wells et al. (1952) with some modifications. Briefly, octanoyl chloride (Sigma Aldrich, Taufkirchen, Germany) was added dropwise to a solution of methyl anthranilate (Sigma Aldrich, Taufkirchen, Germany) dissolved in ethyl acetate at 0°C while stirring. Following heating to 50°C for 5 min the solution was stirred over night at room temperature resulting in a clear solution. Subsequently, this solution was successively washed with water, 1 M sodium hydroxide, 1 M hydrochloric acid and finally with brine. After drying with Na_2_SO_4_ the organic solvent was removed by evaporation leading to a crude oil of methyl *N*-octanoylanthranilate which was used without further purification. Hydrolysis of the acylated anthranilate was carried out with 0.5 M sodium hydroxide solved in ethanol. The reaction mixture was refluxed for 3 h. After cooling, the solution was acidified with 4 M hydrochloric acid and extracted two times with *n*-hexane. Evaporation yielded crude *N*-octanoylanthranilic acid which was recrystallized in *n*-hexane. ESI-TOF/MS for C_15_H_22_NO_3_^+^ ([M + H]^+^): calculated m/z = 264.159; found m/z = 264.114.

CORM-2, a carbon monoxide releasing molecule, and PQS were purchased from Sigma Aldrich (Taufkirchen, Germany).

### Attachment, biofilm formation, and extracellular DNA

Rapid attachment of bacterial cells to a surface was analyzed as described previously (Yeung et al., 2011). Briefly, overnight cultures grown in LB-medium were washed and diluted in LB medium to an OD_600 nm_ of 1.0. Aliquots (100 μl) of this suspension were used to inoculate each well of a microtiter plate. Cells were allowed to adhere for 60 min at 37°C prior to staining with crystal violet. All experiments were done in triplicates with 6 individual repeats per measurement (*n* = 18).

The abiotic solid surface assay was used to measure biofilm formation according to the method described by Friedman and Kolter (2004) with the following modifications. Overnight cultures were diluted 1:100 in fresh LB medium, inoculated into 96-well microtiter plates and incubated for 24 h at 37°C without shaking to allow bacterial adherence and biofilm formation. After incubation the biofilm cells were stained using 0.1% (w/v) crystal violet and the absorbance was measured at 595 nm using a TECAN Infinite® 200 PRO microtiter plate reader.

For the determination of colony forming units (CFU), biofilms were grown in glass bottom petri dishes as described above in the presence or absence of 70 U/ml HodC. After 24 h of incubation, planktonic cells were removed by gentle washing. The remaining, adherent cells were scraped off the petri dish surface using cell scrapers, transferred into a new test tube and vortexed vigorously for 10–30 s. Several dilutions were prepared and the CFU were determined by the drop plate method (Herigstad et al., 2001).

Extracellular DNA in biofilm cultures was determined according to the method described previously (Yang et al., 2007). Briefly, overnight cultures were diluted 1:100 in fresh LB medium supplemented with 0.05 mM propidium iodide and biofilms were grown in microtiter plates at 37°C under static conditions. After 24 h of incubation, the absorbance of propidium iodide was measured at 490 nm using a MultisKan MS photometer (Labsystems, Bradenton, USA) and cell density at 595 nm using a TECAN Infinite® 200 PRO.

### Fluorescence microscopy

For microscopic analyses, overnight cultures of *P. aeruginosa* PA14 *lasB*::Tn were diluted 1:100 in LB, HodC was added at a final concentration of 70 U/ml, and the suspensions were used to inoculate glass bottom petri dishes (MatTek, Ashland, USA). After 24 h of incubation at 37°C under static conditions, planktonic cells were removed from the medium by gently washing with LB medium and the attached viable biofilm cells were stained using 5-cyano-2,3-ditolyl tetrazolium chloride (CTC) for 3 h in the dark as described previously (Li et al., 2013). Fluorescence microscopy was carried out using an Axioplan 2 imaging system (Carl Zeiss, Oberkochem, Germany) with appropriate filter sets.

### Transcriptome analysis

RNA for transcriptome analysis was isolated from biofilm cells of the *P. aeruginosa* PA14 *lasB*::Tn strain, grown for 24 h at 37°C under static conditions in microtiter plates in LB medium in the presence or absence of 70 U/ml HodC or iHodC, respectively. The biofilm cells of 40 wells for each condition were pooled, total RNA was extracted using RNeasy Midi columns (QIAGEN), and DNase treatment of isolated RNA samples were performed as described previously (Breidenstein et al., 2012). Depletion of rRNA was accomplished with the MICROB*Express*™ bacterial mRNA enrichment kit (life technologies) according to the manufacturer's protocol. RNA-Seq was performed as described previously (Tettmann et al., 2014). Briefly, sequencing libraries were generated from 50 ng of rRNA depleted RNA samples following the Truseq RNA protocol (Illumina). Paired end reads (2 × 50 nucleotides) were obtained with a Hiseq1000 using SBS v3 kits (Illumina). Cluster detection and base calling were performed using RTAv1.13, and quality of reads was assessed with CASAVA v1.8.1 (Illumina). The reads were mapped against the genome of *P. aeruginosa* PA14 (accession number: NC_008463) using bowtie2 (Langmead and Salzberg, 2012). The genomic annotation of *P. aeruginosa* PA14 was downloaded from the *Pseudomonas* Genome database (www.pseudomonas.com) (Winsor et al., 2009). Gene expression was determined by counting for each gene the number of reads that overlapped with the annotation location using HTSeq (Anders et al., 2015). Differential expression was calculated using the R package DESeq2 (Love et al., 2014), and genes were assumed to be differentially expressed, if the fold-change was at least two-fold (±) and the *P-value* less than 0.05. Complete expression data is deposited at the Sequence Read Archive NCBI under the accession number SRP046054.

## Results

### HodC is stable in cultures of *P. aeruginosa* PA14 *lasB*::Tn

**Figure 1:**
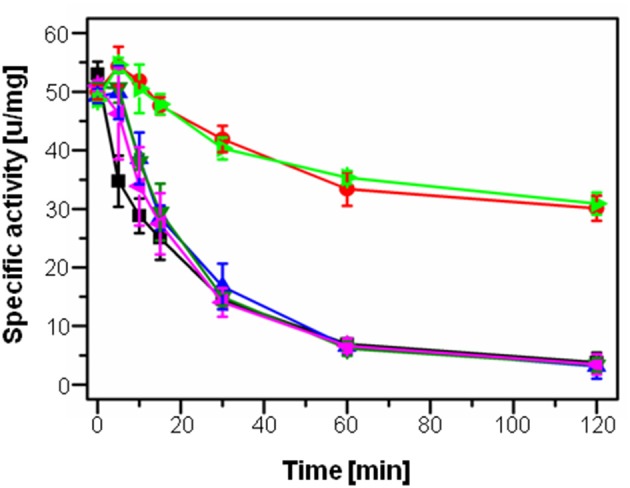
**Specific activity of HodC upon incubation with culture supernatants of ***P. aeruginosa*** strains**. 100 μl/ml sterile LB medium (light green), or 100 μl/ml culture supernatant of *P. aeruginosa* PA14 (blue), the *lasA*::Tn mutant (black), the *aprA*::Tn mutant (dark green), the *prpL*::Tn mutant (pink), and the *lasB*::Tn mutant (red) in sodium phosphate buffer (pH 8.0) were incubated with 0.75 μg/ml HodC at 37°C, and enzyme activity was measured at different time points. Error bars indicate standard deviations from three independent experiments.

The 2-alkyl-3-hydroxy-4(1*H*)-quinolone 2,4-dioxygenase HodC was previously shown to be able to quench PQS signaling in *P. aeruginosa*, leading to decreased production of several virulence factors, however, HodC activity in *P. aeruginosa* cultures was significantly reduced over time by proteolytic cleavage (Pustelny et al., 2009). In order to identify the extracellular protease(s) of *P. aeruginosa* PA14 involved in inactivation of HodC, its activity was determined in the presence of cell-free culture supernatants of *P*. *aeruginosa* PA14 and supernatants of the four protease deficient mutants *lasB*::Tn (inactivation of elastase LasB), *lasA*::Tn (LasA), *prpL*::Tn (protease IV), and *aprA*::Tn (alkaline metalloproteinase). While supernatants of *P. aeruginosa* PA14 as well as of the *lasA, prpL*, and *aprA* mutant strains strongly decreased HodC activity already after 10 min of incubation, no change in activity was observed for supernatants of *P. aeruginosa* PA14 *lasB*::Tn in comparison to the LB control (Figure 1). Therefore, the *lasB* mutant was chosen for all following experiments to ensure high HodC activity even during prolonged incubation periods.

### HodC activity stimulates an increase in biomass in newly formed but also in established biofilms

**Figure 2:**
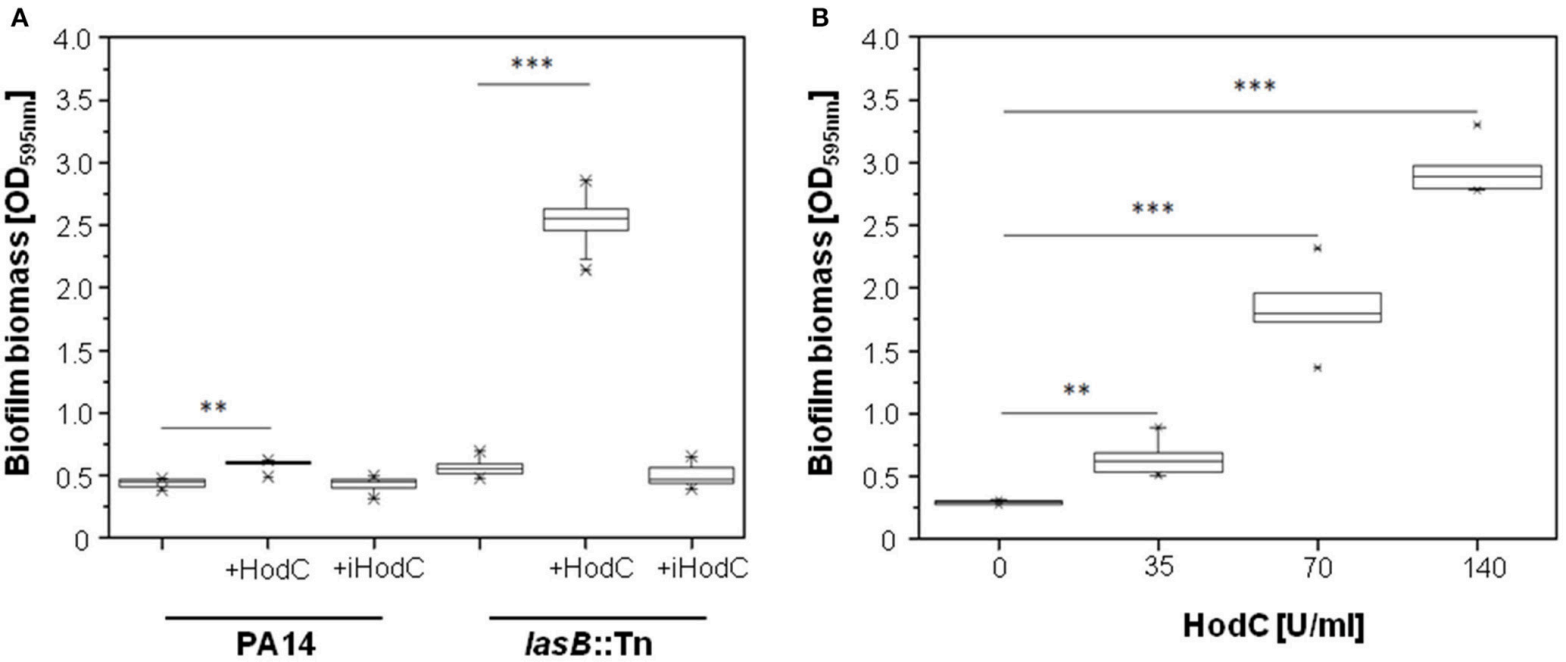
**Effect of HodC on biofilm formation of ***P. aeruginosa***. (A)** Effect of HodC (70 U/ml) and iHodC (at the same protein concentration) on wild-type *P. aeruginosa* PA14 and *P. aeruginosa* PA14 *lasB*::Tn. **(B)** Biofilm formation in response to different concentrations of HodC. **(A,B)** Overnight cultures (in LB) were diluted (1:100) in LB in 96-well microtiter plates, and supplemented with HodC protein as indicated. After incubation for 24 h at 37°C, cells were stained with 0.1% (w/v) crystal violet and quantified by measuring OD_595nm_. Experiments were done in triplicates with 6 individual repeats per measurement (*n* = 18). Statistical analyses were performed with the Mann-Whitney *U*-test. ^**^p ≤ 0.01; ^***^p ≤ 0.001. The highest and lowest outliers are indicated by “X.”

**Figure 3:**
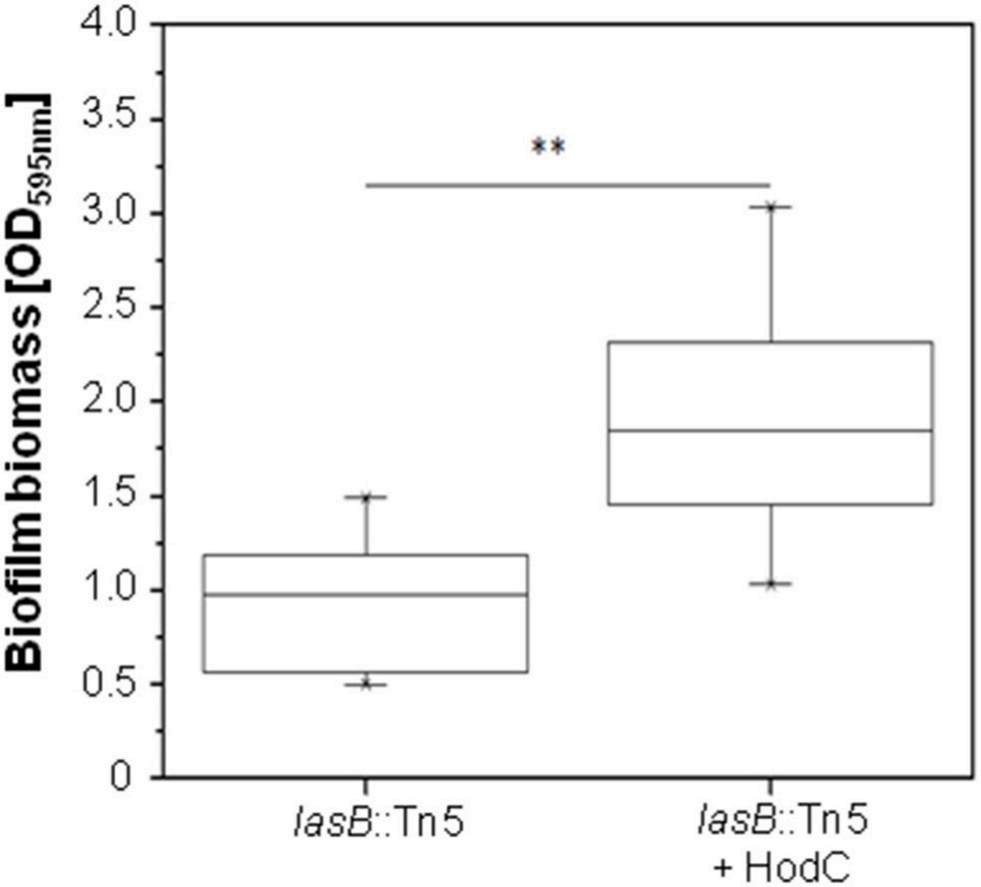
**Effect of HodC on pre-grown biofilms of ***P. aeruginosa*** PA14 ***lasB***::Tn**. Overnight cultures (in LB) were diluted (1:100) in LB in 96-well microtiter plates, and incubated for 24 h at 37°C. After removal of planktonic cells and a washing step with LB, the biofilm was covered with fresh LB without or with HodC (70 U/ml) and incubated for another 24 h at 37°C. Biofilm cells were stained with 0.1% (w/v) crystal violet and quantified by measuring OD_595*nm*_. Experiments were done in triplicates with 6 individual repeats per measurement (*n* = 18). Statistical analyses were performed with the Mann-Whitney *U*-test. ^**^p ≤ 0.01. The highest and lowest outliers are indicated by “X.”

Since PQS is important for biofilm formation (Diggle et al., 2003), we analyzed the effect of HodC on *P. aeruginosa* biofilm development. Addition of HodC to static biofilm cultures of *P. aeruginosa* PA14 resulted in a moderate increase in biofilm biomass after 24 h of incubation, whereas the *lasB*::Tn mutant exhibited a much stronger, five-fold increase in biofilm biomass in the presence of HodC (Figure 2A). This enhancement in biofilm formation increased with increasing concentrations of the enzyme in the culture media (Figure 2B). In contrast, addition of inactive HodC (iHodC) to biofilm cultures of both *P. aeruginosa* PA14 wild-type and the *lasB* mutant did not have an impact on biofilm development (Figure 2A). Remarkably, HodC addition even influenced established biofilms, as we observed an almost two-fold increase of biofilm biomass of pre-grown biofilms in the presence of the enzyme (Figure 3).

**Figure 4:**
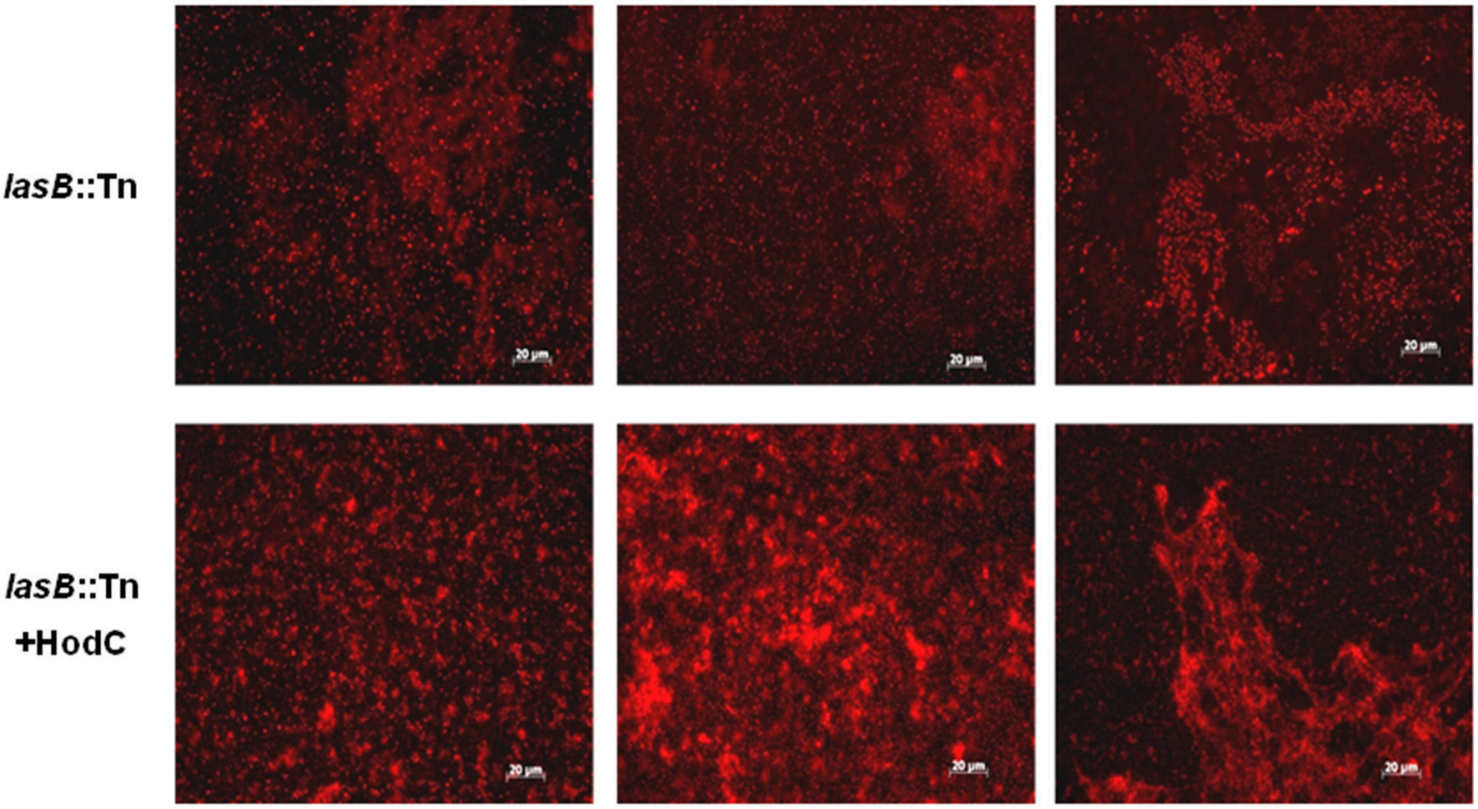
**Biofilms of ***P. aeruginosa*** PA14 ***lasB***::Tn in presence and absence of HodC, stained with CTC**. Overnight cultures were diluted (10^−2^) in LB, supplemented with HodC at a final concentration of 70 U/ml, and the suspensions were used to inoculate glass bottom petri dishes (MatTek, Ashland, USA). After 24 h of incubation at 37°C under static conditions, planktonic cells were removed by washing with LB medium and the attached viable biofilm cells were stained using 5-cyano-2,3-ditolyl tetrazolium chloride (CTC). Fluorescence microscopy was carried out using an Axioplan 2 imaging system with appropriate filter sets. Experiments were performed in triplicate and representative images are shown.

To analyze whether the observed enhancement in biofilm mass was indeed due to an increase in viable cells, we evaluated biofilms of *P. aeruginosa* PA14 *lasB*::Tn on glass bottom petri dishes using fluorescence microscopy in combination with CTC staining, which allows the visualization of metabolically active cells. The biofilms exhibited a much higher proportion of viable cells when grown in the presence of HodC (Figure 4). Moreover, determination of CFUs revealed 9.2 × 10^8^ (± 3 × 10^8^) CFU/ml and 22.5 × 10^8^ (± 2.7 × 10^8^) CFU/ml for untreated and HodC-treated biofilms, respectively, corresponding to a 2.4-fold increase in viable biofilm cells in the presence of HodC. However, HodC (70 U/ml) did not affect planktonic growth of *P. aeruginosa* PA14 in LB (data not shown). Since initial adherence of bacterial cells strongly impacts biofilm development, we investigated whether HodC affects biofilm growth already at the very initial phase of cell attachment. To this end, attachment of *P. aeruginosa* PA14 *lasB*::Tn was determined after 1 h of incubation with or without HodC. Quantification of surface-attached cells by crystal violet staining revealed that HodC addition did not alter initial cell adhesion of *P. aeruginosa* (Supplementary Figure 1A). In addition, quantification of extracellular DNA, which is an important factor in biofilm formation, revealed no differences in eDNA of biofilms grown in the presence of HodC compared to the iHodC control biofilms (Supplementary Figure 1B). Taken together, the results indicate that the enhanced biofilm in presence of HodC is due to the increase in viable biomass.

### The PQS degradation products carbon monoxide and *N*-octanoylanthranilic acid do not influence biofilm development

**Figure 5:**
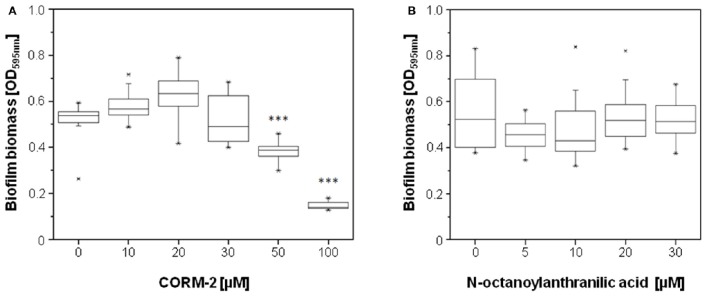
**Effect of the products of HodC-catalyzed PQS cleavage on biofilm formation of ***P. aeruginosa*** PA14 ***lasB***::Tn**. Overnight cultures (in LB) were diluted (10^−2^) **(A)** in BM2 medium (without casamino acids), or **(B)** in LB, and supplemented with the either the CO-releasing molecule CORM-2 **(A)**, or with *N*-octanoylanthranilic acid **(B)** as indicated. After incubation for 24 h at 37°C, cells were stained with 0.1% (w/v) crystal violet and quantified by measuring OD_595*nm*_. Experiments were done in triplicates with 6 individual repeats per measurement (*n* = 18). Statistical analyses were performed with the Mann-Whitney *U*-test. The highest and lowest outliers are indicated by “X.” **(A)** ^***^p ≤ 0.001, in relation to biofilms of *P. aeruginosa* PA14 *lasB*::Tn as formed in absence of compounds.

In order to identify the mechanisms underlying the stimulation in biofilm formation by HodC, we first analyzed whether *N*-octanoylanthranilic acid and carbon monoxide, the products of HodC-catalyzed PQS cleavage, have an effect on biofilm development of the *P. aeruginosa* PA14 *lasB*::Tn strain. In agreement with an earlier study by Murray et al. (2012), no significant increase in biofilm formation was observed in the presence of 10–20 μM of the CO-releasing molecule CORM-2, but biofilm formation was decreased strongly at higher concentrations of CORM-2 (Figure 5A). Given that supernatants of overnight cultures of *P. aeruginosa* PA14 contain between 5 and 25 μM PQS (Diggle et al., 2003; Lépine et al., 2003), we analyzed the impact of *N*-octanoylanthranilic acid on biofilm biomass at concentrations of up to 30 μM. The data (Figure 5B) indicated that this PQS cleavage product has also no influence on biofilm development. Overall, these results indicated that the two metabolites formed by HodC-mediated PQS cleavage are not responsible for the observed biofilm phenotype.

### HodC activity in biofilm cultures results in down-regulation of fur-controlled genes, likely due to increased iron availability

To get an insight into the molecular basis for the observed biofilm phenotype, transcriptome analyses were performed using Illumina sequencing, and the expression profile of *P. aeruginosa* PA14 *lasB*::Tn biofilms treated with HodC was compared to that of untreated control biofilm cells. This analysis identified a total set of 38 genes that were differentially regulated in the presence of HodC (Table 2). Most of the 34 genes that were down-regulated are directly or indirectly under control of the ferric uptake regulator Fur (Ochsner et al., 2002). Among them are the operon comprising the fumarate hydratase (*fumC*) and superoxide dismutase (*sodM*) genes (Hassett et al., 1997), and genes coding for the regulation and biosynthesis of the siderophore pyoverdine (*pvdS, pvdL, pvdA, pvdQ*) and for proteins involved in heme uptake and degradation (*phuS, phuR, hemO*). Additional transcriptome analyses for cells incubated with iHodC did not reveal any significant differences in gene expression except a 3.1-fold upregulation of the *glpD* gene, which is coding for glycerol-3-phosphate dehydrogenase, in comparison to the untreated control biofilms (data not shown). This *glpD* upregulation was comparable to the expression observed in response to HodC (Table 2) and is most likely due to the presence of glycerol in the enzyme stock solutions. Thus, the observed transcriptome data demonstrate that the changes in gene expression are entirely depending on the catalytic activity of HodC.

**Table 2:** **Genes of ***P. aeruginosa*** PA14 ***lasB***::Tn with decreased expression in presence of HodC**.

**PA number**	**PA14 locus tag**	**Fold change**	**Gene name**	**Gene product**	**Regulator binding sites in operator region**
n.a.	PA14_54870	−6.0		Hypothetical protein	
PA0672	PA14_55580	−5.8	*hemO*	Heme oxygenase	Fur box^a^
PA1300	PA14_47400	−8.4	–	RNA polymerase ECF-subfamily sigma-70 factor	Fur box^a^
PA2033	PA14_38220	−9.1	–	Hypothetical protein	Fur box^a^
PA2204	PA14_36200	−5.4	–	Probable binding protein component of ABC transporter	
PA2331	PA14_34460	−3.7	–	Hypothetical protein	
PA2385	PA14_33820	−6.3	*pvdQ*	3-Oxo-C12-homoserine lactone acylase PvdQ	Fur box^a^
PA2386	PA14_33810	−8.3	*pvdA*	L-ornithine N5-oxygenase	PvdS binding site^a^
PA2393	PA14_33730	−5.0	–	Putative dipeptidase	PvdS binding site^a^
PA2397	PA14_33690	−4.4	*pvdE*	Pyoverdine biosynthesis protein PvdE	PvdS binding site^a^
PA2398	PA14_33680	−6.9	*fpvA*	Ferripyoverdine receptor	PvdS binding site^a^
PA2399	PA14_33650	−3.5	*pvdD*	Pyoverdine synthetase D	PvdS binding site^a^
PA2400	PA14_33630	−3.4	*pvdJ*	PvdJ	PvdS binding site^a^
PA2402	PA14_33610	−3.9		Probable non-ribosomal peptide synthetase	PvdS binding site^a^
PA2412	PA14_33510	−5.8		Conserved hypothetical protein	Operon 2411-2412: PvdS binding site^a^
PA2424	PA14_33280	−7.9	*pvdL*	Peptide synthase PvdL	PvdS binding site^a^
PA2426	PA14_33260	−7.0	*pvdS*	RNA polymerase ECF-subfamily sigma-70 factor PvdS	Fur box^a^
PA3049	PA14_24650	−3.0	*rmf*	Ribosome modulation factor	
PA3519	PA14_18810	3.2	–	Hypothetical protein	CueR binding site^b^,
PA3522	PA14_18780	3.7	*mexQ*	Probable RND efflux transporter	Operon 3523-3521: CueR binding site^b^
PA3523	PA14_18760	4.5	*mexP*	Probable RND efflux membrane fusion protein precursor	
PA3530	PA14_18680	−3.0	*bfd*	Bacterioferritin-associated ferredoxin	Fur box^a^
PA3584	PA14_17930	3.4	*glpD*	Glycerol-3-phosphate dehydrogenase	GlpR binding site^c^
PA3790	PA14_15070	−4.4	*oprC*	Putative copper transport outer membrane porin OprC precursor	
PA4156	PA14_10200	−6.9	*fvbA*	Ferric vibriobactin receptor FvbA	Fur box^d^
PA4221	PA14_09340	−4.4	*fptA*	Ferric pyochelin outer membrane receptor precursor	Operon PA4220-4221; Fur box^a^
PA4228	PA14_09240	−4.0	*pchD*	Pyochelin biosynthesis protein PchD	Putative Fur binding site^e^
PA4467	PA14_57990	−9.3		Hypothetical protein	Operon PA4467-4471; Fur box^a^
PA4468	PA14_58000	−12.2	*sodM (sodA)*	Manganese superoxide dismutase	
PA4469	PA14_58010	−8.7	–	Hypothetical protein	
PA4470	PA14_58030	−18.1	*fumC1*	Fumarate hydratase	
PA4471	PA14_58040	−8.4	–	Hypothetical protein	
PA4570	PA14_60480	−22.1	–	Hypothetical protein	Fur box^a^
PA4708	PA14_62300	−5.1	*phuT*	Heme-transport protein, PhuT	Operon 4709-4705: Fur box^a^
PA4709	PA14_62330	−5.6	*phuS*	Heme-degrading enzyme PhuS	
PA4710	PA14_62350	−4.5	*phuR*	Heme/hemoglobin uptake outer membrane receptor PhuR precursor	Fur box^a^
PA4896	PA14_64700	−7.0	–	Probable RNA polymerase ECF-subfamily sigma-70 factor	Fur box^a^
PA4704.1	n.a.	−4.1	*prrF1*	Small RNA PrrF1	Fur box^f^

a *Ochsner et al. (2002)*.

b *Thaden et al. (2010)*.

c *Schweizer and Po (1996)*.

d *Elias et al. (2011)*.

e *www.pseudomonas.com*.

f *Wilderman et al. (2004)*.

**Figure 6:**
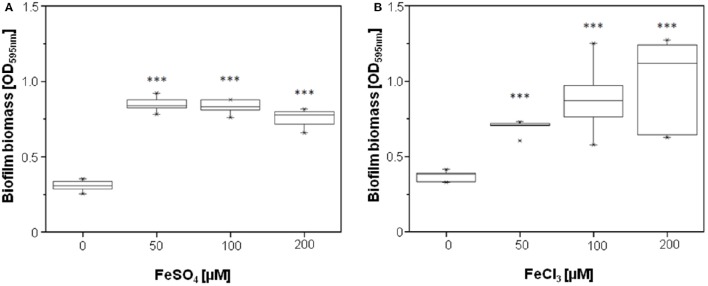
**Effect of FeSO_**4**_ and FeCl_**3**_ on biofilm formation of ***P. aeruginosa*** PA14 ***lasB***::Tn**. Overnight cultures (in LB) were diluted (10^−2^) in BM2 medium, and supplemented with FeSO_4_ **(A)** or FeCl_3_ **(B)** as indicated. After incubation for 24 h at 37°C, cells were stained with 0.1% (w/v) crystal violet and quantified by measuring OD_595*nm*_. Experiments were done in triplicates with 6 individual repeats per measurement (*n* = 18). Statistical analyses were performed with the Mann-Whitney *U*-test. The highest and lowest outliers are indicated by “X.” ^***^p ≤ 0.001.

Because PQS exhibits iron-chelating properties and therefore sequesters iron from the culture medium (Bredenbruch et al., 2006; Diggle et al., 2007), we assumed that the observed downregulation of genes involved in iron metabolism by HodC is due to an increase in available iron in the culture medium as a result of PQS cleavage. This hypothesis is supported by the finding that in chemically defined medium, biofilm formation of the *P. aeruginosa lasB* mutant was significantly enhanced by the addition of 50 μM FeSO_4_ or FeCl_3_ (Figure 6).

## Discussion

Interference with bacterial quorum sensing systems by inactivation of signal molecules has been discussed as a promising anti-virulence strategy, because the signal can hardly become unsusceptible to the quorum quenching enzyme without losing its activity, and because a quorum quenching agent addressing an extracellular target may exert less selection pressure than an agent that acts intracellularly (García-Contreras et al., 2013). However, some drawback of using enzymes to inactivate signal molecules is their susceptibility to denaturation by adverse physicochemical conditions and to proteolytic degradation. In *P. aeruginosa*, LasB elastase represents one of the major secreted proteins, participating in the proteolytic inactivation of numerous extracellular components of the innate and adaptive immune system of the eukaryotic host (Leduc et al., 2007). LasB also was found to be the basic cause of rapid inactivation of the PQS-cleaving dioxygenase HodC in *P. aeruginosa* cultures. Thus, to be applicable as a quorum quenching enzyme, the protein needs to be stabilized by enzyme engineering, or protected by immobilization or encapsulation.

HodC-mediated inactivation of PQS quenches the production of virulence factors such as pyocyanin, rhamnolipids, and lectinA, whose production is upregulated in response to PQS signaling (Pustelny et al., 2009). However, HodC-catalyzed cleavage of PQS turned out to stimulate *P. aeruginosa* biofilm development. This stimulatory effect appears to be due to the fact that PQS cleavage removes not only a signal, but also an iron trap. PQS has been reported to form 2:1 and 3:1 chelate complexes with ferric ions (Diggle et al., 2007). The transcriptome profile of *P. aeruginosa* in response to PQS revealed a marked induction of genes related to iron acquisition and oxidative stress response; for most genes this differential regulation was due to the iron-chelating effect of PQS (Bredenbruch et al., 2006). Considering that fresh LB medium contains about 6 μM iron (Diggle et al., 2007) and PQS levels (at least in in planktonic cultures) can reach up to ~25 μM, PQS likely induces a limitation of readily bioavailable iron ions.

Both iron limitation and iron excess can adversely affect biofilm formation in *P. aeruginosa*, with iron excess promoting biofilm dispersal (Singh et al., 2002; Singh, 2004; Banin et al., 2005; Musk et al., 2005; Yang et al., 2007; Patriquin et al., 2008). Interestingly, growth yields of *P. aeruginosa* biofilm were reported to be affected to a greater extent by iron limitation than planktonic *P. aeruginosa* cultures (Patriquin et al., 2008). The HodC-mediated increase in viable biofilm biomass as well as the downregulation of a set of iron-controlled genes is in line with the notion that the enzyme-catalyzed PQS cleavage increases iron availability. Under the conditions tested, the growth-promoting effect of iron, released from the PQS-iron complex by degradation of the PQS ligand, overrides the HodC-mediated quenching of the stimulatory effect PQS may have on biofilm development. Thus, under conditions of iron limitation, degradation of PQS is contraindicated for combating *P. aeruginosa* PA14 biofilms.

## Author contributions

JO, SF, and GB conceived the experiments. CN purified HodC proteins and performed enzyme activity and stability assays. BT and AN performed biofilm experiments and gene expression profiling. AD analyzed the Illumina sequencing data and FK performed the chemical synthesis of *N*-octanoylanthranilic acid. JO and SF wrote the paper. All authors contributed to the final version of the manuscript, and all authors approved the final manuscript.

## Funding

We gratefully acknowledge financial support by the BioInterfaces in Technology and Medicine (BIFTM) Program of the Karlsruhe Institute of Technology (KIT) in the Helmholtz Association, and support by the Deutsche Forschungsgemeinschaft within GRK1409 and grant no. FE 383/25-1.

### Conflict of interest statement

The authors declare that the research was conducted in the absence of any commercial or financial relationships that could be construed as a potential conflict of interest.
