# Supplementary material for: Enzyme-Mediated Quenching of the Pseudomonas Quinolone Signal (PQS) Promotes Biofilm Formation of Pseudomonas aeruginosa by Increasing Iron Availability
Source: Front Microbiol. 2016 Dec 9;7:1978. doi: 10.3389/fmicb.2016.01978 (PMC5145850; doi:10.3389/fmicb.2016.01978)
Supplement: Supplementary file 1 [file DataSheet1.PDF]

Supplementary Material

Enzyme-mediated quenching of the *Pseudomonas* quinolone signal (PQS) promotes biofilm formation of *Pseudomonas aeruginosa* by increasing iron availability

Beatrix Tettmann, Christine Niewerth, Frank Kirschhöfer, Anke Neidig, Andreas Dötsch, Gerald Brenner-Weiss, Susanne Fetzner, and Joerg Overhage*

*** Correspondence:** Corresponding Author: joerg.overhage@kit.edu

1.1 Supplementary Figure

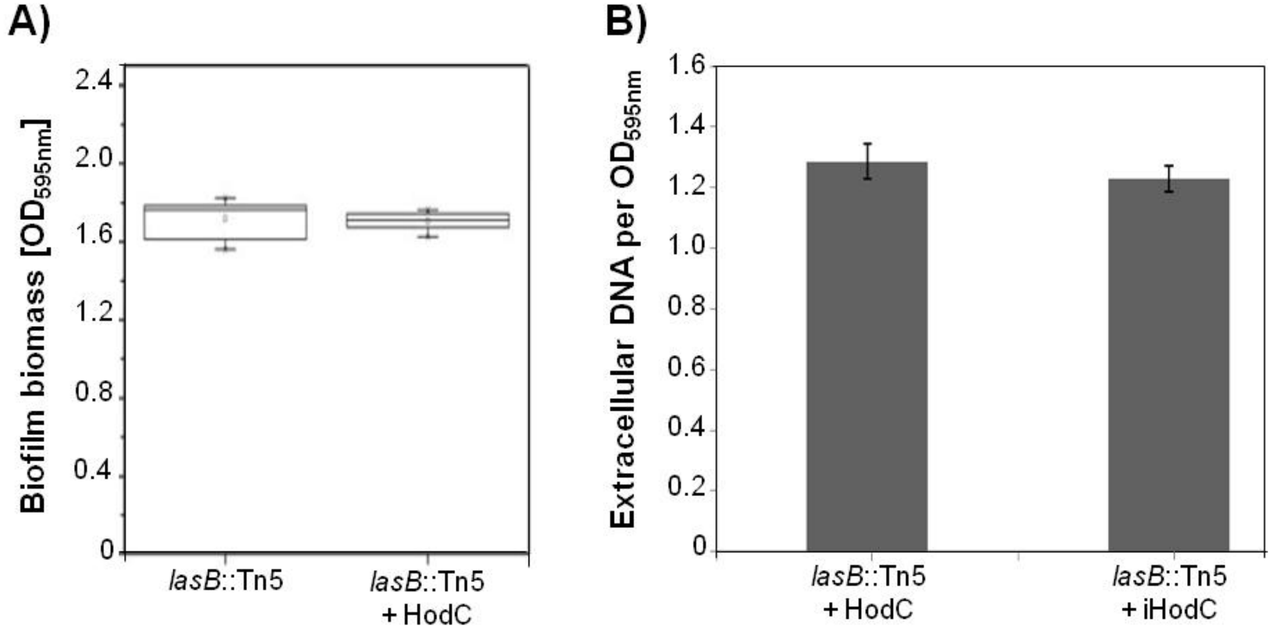

Supplementary Figure 1. Effect of HodC on attachment and extracellular DNA release of *P. aeruginosa* PA14 *lasB*::Tn. (A) Effect of HodC on initial attachment of *P. aeruginosa* PA14 *lasB*::Tn. Overnight cultures (in LB) were washed and diluted in LB in 96-well microtiter-plates to an OD_{600nm} of 1.0, and supplemented with HodC protein (70 U/ml). Cells were allowed to adhere for 60 min at 37 °C prior to staining with 0.1 % (w/v) crystal violet and quantification by measuring OD_{595nm}. Experiments were done in triplicates with 6 individual repeats per measurement (n=18). (B) Effect of HodC on extracellular DNA in biofilms of *P. aeruginosa* PA14 *lasB*::Tn. Overnight cultures were diluted 1:100 in fresh LB medium supplemented with 0.05 mM propidium iodide and biofilms were grown in microtiter plates at 37 °C, and supplemented with HodC (70 U/mg) or iHodC protein (at the same protein concentration). After 24 hours of incubation, relative amounts of extracellular DNA in biofilms were determined by measuring the absorbance of propidium iodide at OD_{490nm} and cell density at OD_{595nm}. Experiments were done in triplicates with 2 individual repeats per measurement (n=6). Statistical analyses for (A) and (B) were performed with the Mann-Whitney U test.
